# Effects of Changes in Ankle Joint Angle on the Relation Between Plantarflexion Torque and EMG Magnitude in Major Plantar Flexors of Male Chronic Stroke Survivors

**DOI:** 10.3389/fneur.2020.00224

**Published:** 2020-04-07

**Authors:** Jongsang Son, William Zev Rymer

**Affiliations:** ^1^Shirley Ryan AbilityLab (formerly the Rehabilitation Institute of Chicago), Chicago, IL, United States; ^2^Department of Physical Medicine and Rehabilitation, Northwestern University, Chicago, IL, United States

**Keywords:** EMG-torque relation, stroke, muscular contraction efficiency, force-length relation, muscle weakness

## Abstract

The slope of the EMG-torque relation is potentially useful as a parameter related to muscular contraction efficiency, as a greater EMG-torque slope has often been reported in stroke-impaired muscles, compared to intact muscles. One major barrier limiting the use of this parameter on a routine basis is that we do not know how the EMG-torque slope is affected by changing joint angles. Thus, the primary purpose of this study is to characterize the EMG-torque relations of triceps surae muscles at different ankle joint angles in both paretic and non-paretic limbs of chronic hemispheric stroke survivors. Nine male chronic stroke survivors were asked to perform isometric plantarflexion contractions at different contraction intensities and at five different ankle joint angles, ranging from maximum plantarflexion to maximum dorsiflexion. Our results showed that the greater slope of the EMG-torque relations was found on the paretic side compared to the non-paretic side at comparable ankle joint angles. The EMG-torque slope increased as the ankle became plantarflexed on both sides, but an increment of the EMG-torque slope (i.e., the coefficient *a*) was significantly greater on the paretic side. Moreover, the relative (non-paretic/paretic) coefficient *a* was also strongly correlated with the relative (paretic/non-paretic) maximum ankle plantarflexion torque and with shear wave speed in the medial gastrocnemius muscle. Conversely, the relative coefficient *a* was not well-correlated with the relative muscle thickness. Our findings suggest that muscular contraction efficiency is affected by hemispheric stroke, but in an angle-dependent and non-uniform manner. These findings may allow us to explore the relative contributions of neural factors and muscular changes to voluntary force generating-capacity after stroke.

## Introduction

Weakness of voluntary muscle contraction is a dominant clinical feature after hemispheric stroke, and the severity of such weakness is correlated with a stroke survivor's independence in performing many functional tasks (1). This reduction in voluntary muscle strength is typically the most obvious motor deficit (2), and many clinical phenomena observed in chronic stroke survivors could be attributed to a loss of strength rather than to a loss of control (3).

In addition to muscle weakness, muscular contractions in stroke-impaired muscles often appear less efficient than in contralateral muscles or in analogous muscles of intact subjects, potentially contributing to impaired voluntary force generation. To illustrate this assertion further (4), reported that in approximately half of the tested human stroke survivors, during sustained isometric voluntary contractions at different intensities, the slope of the biceps brachii (BB) electromyogram (EMG)-force relations was significantly greater on the paretic side than on the non-paretic side. A similar finding was also observed in paretic first dorsal interosseous (FDI) muscles of chronic stroke survivors (5), implying that paretic muscles may require the recruitment of more motor units to achieve a given muscle force, and thus, display inefficient muscular contractions.

It is evident then, that altered motor unit behavior may result in inefficient muscular contractions. For example, abnormally low mean motor unit firing rates were observed in paretic tibialis anterior (TA) muscles (6, 7) and in paretic BB muscles (8). There is also evidence for additional altered motor unit behavior, including compressed motor unit recruitment threshold ranges during isometric contractions of the paretic BB muscles (8), impairments in firing rate modulation in paretic BB muscles (9), disorganization in the rank order of recruitment in paretic FDI muscles (10), and a compressed range of motor unit firing rates in paretic FDI muscles (11). Moreover, a recent simulation study revealed that the recruitment compression and a compressed “onion-skin” firing pattern can potentially also contribute to voluntary muscle weakness (12). In addition, the slope of EMG-force relation was reported to increase in concert with compressed motor unit recruitment threshold ranges and reductions of mean motor unit firing rates (13). Although these earlier studies support the idea that altered neural factors can contribute, these neural factors may not be enough to explain a complicated EMG-force relation, because the EMG-force relation is an outcome of both neural and muscular factors.

In particular, muscular changes may also contribute to voluntary muscle weakness (14–17). Although our understanding of muscular changes after hemispheric stroke is still limited, it is relevant to note that decreased muscle thickness is often associated with reduced force output at a given level of muscle activation. In addition, the maximum isometric muscle force varies depending on the muscle length (18), and this force-length relation may also be substantially altered after stroke. For example, the width of the active force-length curve seems narrower in the paretic medial gastrocnemius (MG) muscles (19) than in the equivalent contralateral muscles. Such altered contractile properties may lead to modified torque-angle curves, showing a significant reduction in the magnitude of the torque at joint angles where muscle length is short (3, 20–22), potentially resulting in a higher slope of the EMG-torque relation at such a short length. This outcome is likely because the effective torque at shorter lengths is smaller at a given EMG. Furthermore, material properties of muscle tissues seem to contribute to muscle mechanics, revealing that a stiffer material surrounding contractile elements of muscle tissues can reduce fascicle strain as well as muscle force (23–26). Based on these observations, it is likely that muscular factors can also play a part in the abnormal EMG-torque relation observed in chronic stroke survivors.

In light of these uncertainties, the purpose of this study is to characterize the EMG-torque relations of calf muscles at different ankle joint angles on both paretic and non-paretic sides in chronic stroke survivors, and thus, to understand how the slope of such relations (i.e., muscle efficiency-related parameter) is altered by changing joint angles. We hypothesize that the slope becomes greater as calf muscle lengths shorten because the operating range of calf muscles is usually located in the ascending limb of active force-length curve, so that the maximum force gradually decreases with shortening muscle lengths (27–29). We also hypothesize that as the muscle length becomes shorter, a greater increase in the slope is more likely to be observed on the paretic side than on the non-paretic side. This is because the width of the active force-length curve may be narrower on the paretic side (19) and thus, the relative reduction in the magnitude of peak forces on the paretic side becomes greater at comparable muscle lengths.

As a secondary goal, we also seek to characterize the relation between the maximum joint torque at each joint angle and the EMG-torque slope at this joint angle. Our hypothesis here is that the greater the slope, the smaller the maximum joint torque. This is because the slope is a measure of muscular contraction efficiency.

In order to better understand the potential impact of intrinsic muscular changes of muscular contraction efficiency after, we propose to assess associations between the relative slope-related parameter and the relative shear wave speed (SWS) and between the relative slope-related parameter and the relative muscle thickness. SWS is a non-invasive measure of tissue stiffness (30), and it has been shown that shear waves travel faster in paretic MG muscles than in non-paretic muscles of chronic stroke survivors (31, 32).

## Methods

### Participants

Nine male chronic stroke survivors participated in this study (age: 56.9 ± 7.8 yrs.; height: 174.8 ± 7.3 cm; weight: 81.1 ± 8.9 kg; time since stroke: 8.1 ± 4.1 yrs.). Inclusion criteria were: (1) chronic stroke survivors (more than 6 months after stroke); (2) age 18–75 years; and (3) medically stable with medical clearance to participate. Exclusion criteria were: (1) other physical impairments such as orthopedic injuries or recent surgeries in lower limbs; (2) knee or ankle contractures >10°; (3) unstable neurological or cardiovascular conditions that may affect the candidate's performance; (4) cognitive limitations, such that the subject cannot follow instructions; (5) anti-spasticity drug injections (such as botulinum toxin) in the 3 months prior to participation; and (6) musculoskeletal pain that interferes with participation in this study. All participants were ambulating independently and were not currently receiving physical therapy. They had no history of botulinum toxin treatments for at least 6 months before testing. Written informed consent was obtained from all participants prior to testing and Northwestern University's Institutional Review Board approved all procedures.

### Experimental Setup

Participants were seated upright in a fully-adjustable chair with the trunk and thigh firmly strapped to the chair. The foot was secured to the footplate where a 6-axis force-measuring sensor (Omega160, ATI Industrial Automation, Apex, NC, USA) was installed. The knee joint was extended as much as possible to a comfortable position. The ankle center of rotation (defined as the mid-line between the malleoli) was aligned with the rotation axis of the dynamometer. A slight adjustment in the subject's posture was then allowed to make sure the subject was comfortable throughout the experiment (in most cases, the knee was flexed ~10°).

Single differential surface EMG (sEMG) electrodes (GRASS, Asto-Med, Inc., West Warwick, Rhode, Island) were placed over the muscle belly of the MG, the lateral gastrocnemius (LG), the soleus (SOL), and the tibialis anterior (TA), in order to record activity in each muscle. A ground electrode was attached to the patella. The area for the electrodes was cleaned with alcohol pads before electrode placement.

A shear wave elastography ultrasound system (Aixplorer Supersonic Imagine, Aix-en-Provence, France) with a linear transducer array (4–15 MHz, SuperLinear SL15-4, Vermon, Tours, France) (30) was used to record the MG muscle thickness and SWS under passive conditions at each designated angle. The transducer was positioned directly adjacent to the EMG electrode of the MG muscle, avoiding interference between the transducer and the EMG electrode, and was then secured to the shank using a custom holder, in order to minimize translation, and pressure induced by the transducer.

During the experiment, voluntary isometric plantarflexion torque and EMG signals were recorded at a sampling rate of 2 kHz and synchronized through a data acquisition (DAQ) system (NI USB-6259 BNC, National Instrument, Austin, TX, USA).

### Procedures

To investigate the effects of changing ankle joint angle on the slope of EMG-torque relations, five different ankle angles were chosen: maximum plantarflexion (PF), maximum dorsiflexion (DF), neutral, and two intermediate angles. The maximum PF and DF positions were determined while the ankle joint was passively moved by the experimenter. Then, two intermediate angles were set as angles between maximum DF and neutral or maximum PF and neutral. Positive angles indicate dorsiflexion and negative angles plantarflexion.

At each designated ankle angle, and in a randomized order, ultrasound images from the MG muscle were captured while the muscle was relaxed. The region of interest (RoI) for SWS measurements was manually located over the muscle belly. Subjects were then asked to perform maximum voluntary isometric contractions (MVICs) in ankle plantarflexion for 5 s each, with a 1 min break between each MVC trial. The average value of three maximum MG muscle activations for each MVC trial was used to calculate the level of the MG muscle contraction intensity for visual feedback. The subject was then asked to perform sustained isometric plantarflexion contractions at four different contraction levels (i.e., 0, 20, 40, and 60%MVIC) for 5 s. Three isometric plantarflexion contractions were elicited at each designated contraction level randomized. A 30 s rest period between repetitions was provided. Two sessions were separately conducted for both paretic and non-paretic sides.

### Data Analysis

Torque signals were processed by applying a zero-phase second-order Butterworth low pass filter with a cut-off frequency of 6 Hz, followed by the root mean square (RMS) envelope with a moving window of 500 ms. The maximum isometric plantarflexion torque was determined using peak RMS envelope values corresponding to three MVC trials. EMG signals were processed by applying a zero-phase second-order Butterworth bandpass filter (bandwidth: 20–450 Hz), followed by the RMS envelope with the same moving window, in order to obtain RMS EMG values. The average value of the 1-s segment in each trial was estimated to represent the RMS EMG and ankle plantarflexion torque. This segment was determined by the minimum variability (i.e., standard deviation) of the 1-s window of each processed torque signal.

**Figure 1:**
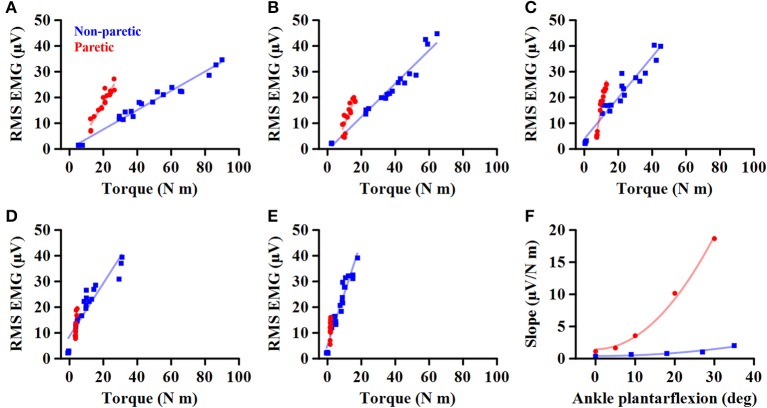
Representative EMG-torque relations at different ankle joint angles from maximum dorsiflexion to maximum plantarflexion **(A–E)**. Overall, the EMG-torque slope on the paretic side (in blue) is greater than on the non-paretic side (in red). Moreover, the more the ankle plantarflexion, the greater the EMG-torque slope. **(F)** Greater changes in the EMG-torque slope on the paretic side with increasing ankle plantarflexion angle.

Afterward, the EMG-torque relations for MG, LG, and SOL muscles were derived, using the RMS EMG and the torque values. Considering the load-sharing among the calf muscles for the ankle joint, the arithmetic mean values of the calf muscles' RMS EMG (ALL) were calculated and then used to characterize the EMG-torque relation. A linear fitting was conducted to determine the slope of the EMG-torque relations (Figures 1A–E).

To determine how the EMG-torque slope would be affected by changes in joint angle and by the stroke, the relationship between the slope and the joint angle was determined as *S* = *aA*^2^ + *b*, where *S* is the slope, *A* the joint angle, *a* the scaling factor, and *b* the intercept. As the slope was monotonically increased with increasing ankle plantarflexion, the maximum DF angle was subtracted by the joint angle (i.e., shift all the joint angle data to start from zero), so that the intercept *b* indicates the slope at the maximum DF angle. The greater coefficient *a* indicates the more increment in the slope, as the ankle joint becomes plantarflexed (Figure 1F).

**Figure 2:**
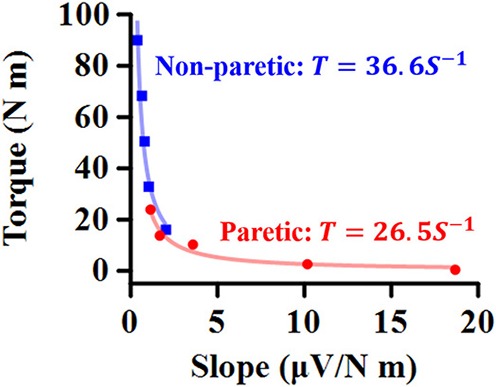
Representative relationship between maximum torque (*T*) at each ankle angle and EMG-torque slope (*S*) at the corresponding angle. The smaller coefficient value, the quicker decay in *T* as *S* increases.

The relation between the maximum joint torque (*T*) at each joint angle and the slope (*S*) at corresponding angles was characterized as *T* = *cS*^−1^, where *c* is the shape coefficient. The smaller coefficient *c*, the quicker decay in the torque as the slope increases (Figure 2).

Image processing was performed to obtain the MG muscle thickness and SWS of the MG muscle. The MG muscle thickness was measured as the distance between superficial and deep aponeuroses (33). The RGB-colored map in RoI of each image was converted into SWS, and the average value of SWS over the largest muscular region was calculated for each image (34). All the data processing was performed, using custom-written software in MATLAB (Mathworks, Natick, USA).

### Statistical Analysis

The RMS EMG and the maximum joint torque were compared between sides using repeated-measures ANOVA. Within-subject factors were the side (i.e., paretic or non-paretic) and the ankle joint angle. Multiple comparisons were done with Bonferroni adjustment. IBM SPSS Statistics (Version 21, IBM, New York, USA) with the significance level (α) of 0.05. The partial eta squared ($\eta_{p}^{2}$) was used to report the effect size for each comparison.

Further statistics were tested with a significance level α < 0.05, using custom-written software in MATLAB (Mathworks, Natick, USA). The Kolmogorov-Smirnov test was performed to assess the assumption of the normal distribution for the fitting parameters (i.e., the coefficient *a, b*, and *c*) as well as ankle joint range of motion. As all the variables did not satisfy the assumption (*p* < 0.05), the Wilcoxon signed-rank test was conducted to evaluate the paired difference in the variables between the paretic and non-paretic sides. Cohen's *d*_*z*_ (35) was then calculated for the standardized mean difference effect size for within-subjects design (36). The data were described as the median value and the interquartile range (IQR).

The Spearman ranked correlation coefficient was calculated to determine the relationship between the relative (i.e., non-paretic/paretic) coefficient *a* and the relative (i.e., paretic/non-paretic) maximum plantarflexion torque, the relative SWS, or the relative muscle thickness. As the coefficient *a* value was greater on the paretic side, the relative coefficient *a* was calculated as the ratio of the non-paretic to the paretic values and thus, the smaller relative coefficient *a* indicates the less efficient muscular contraction on the paretic side compared to the non-paretic side.

## Results

The total number of subjects examined was 9 for MG and LG analyses and 8 for SOL and ALL analyses. This is because we excluded the subject when reporting SOL and ALL as the EMG recording from the SOL muscle was not successful in one of our subjects.

### Passive Range of Motion at Ankle Joint

The passive range of motion at ankle joint on the paretic side (32.0°; IQR = 30.0–40.0) was significantly smaller by ~25% than on the non-paretic side (42.0°, IQR = 37.3–45.0) (*p* = 0.031, *d*_*z*_ = 0.992). Although the median maximum PF (30.0°, IQR = 30.0–35.3) and DF (−3.0°, IQR = −6.3–1.5) on the paretic side was smaller than on the non-paretic side (PF: 34.0°, IQR = 30.0–37.8; DF: −5.0°, IQR = −11.3 to −3.8), there was no significant side-to-side difference in the maximum PF (*p* = 0.156, *d*_*z*_ = 0.548) or in the maximum DF (*p* = 0.063, *d*_*z*_ = 0.816).

### Effect of Joint Angles on Maximum RMS EMG and Joint Torque

**Figure 3:**
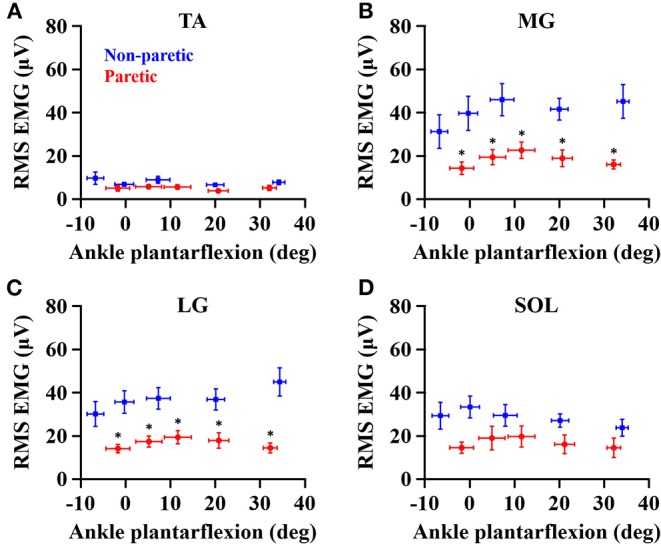
Maximum RMS EMG in tibialis anterior **(A)**, medial gastrocnemius **(B)**, lateral gastrocnemius **(C)**, and soleus **(D)** muscles at each ankle angle. Negative angles indicate dorsiflexion. *a significant difference in pairwise comparisons between the side (*p* < 0.05).

When compared to the non-paretic side, the maximum RMS EMG on the paretic side was significantly smaller in the MG (*p* = 0.003, $\eta_{p}^{2}$ = 0.683) and LG (*p* = 0.003, $\eta_{p}^{2}$ = 0.685) muscles, but not in the TA (*p* = 0.122, $\eta_{p}^{2}$ = 0.272) and SOL (*p* = 0.096, $\eta_{p}^{2}$ = 0.307) muscles (Figure 3). However, the maximum RMS EMG was not a function of the ankle joint angles in all the muscles (TA: *p* = 0.104, $\eta_{p}^{2}$ = 0.734; MG: *p* = 0.093, $\eta_{p}^{2}$ = 0.747; LG: *p* = 0.366, $\eta_{p}^{2}$ = 0.521; SOL: *p* = 0.172, $\eta_{p}^{2}$ = 0.666). Moreover, there was no significant interaction between the side and the ankle joint angle in all the muscles (TA: *p* = 0.387, $\eta_{p}^{2}$ = 0.507; MG: *p* = 0.271, $\eta_{p}^{2}$ = 0.586; LG: *p* = 0.247, $\eta_{p}^{2}$ = 0.604; SOL: *p* = 0.427, $\eta_{p}^{2}$ = 0.481). Note that the magnitude of TA RMS EMG was considerably smaller than the other muscles, which indicates that the potential effects of muscle co-contraction would likely be negligible.

**Figure 4:**
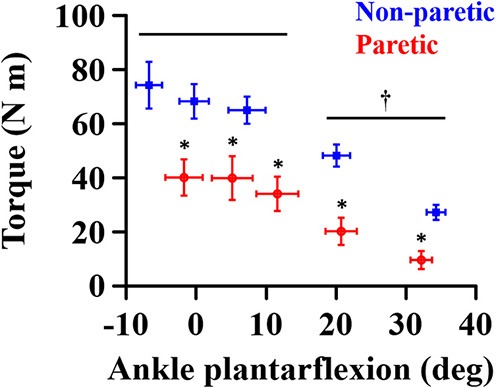
Maximum joint torque at each ankle angle. Negative angles indicate dorsiflexion. ^*^a significant difference in pairwise comparisons between the side (*p* < 0.01). ^†^a significant difference in torque between dorsiflexed angles and plantarflexed angles (*p* < 0.05).

The overall magnitude of the maximum joint torque on the paretic side was significantly smaller than on the contralateral side (*p* = 0.001, $\eta_{p}^{2}$ = 0.776; Figure 4), showing significant differences at all the angles (*p* < 0.01). Furthermore, the maximum joint torque increased as the ankle joint became dorsiflexed (*p* = 0.029, $\eta_{p}^{2}$ = 0.845), showing that the maximum joint torque at the dorsiflexed angles beyond the neutral angles was significantly greater than at the plantarflexed positions (*p* < 0.05). There was a significant interaction between the side and the ankle joint angle (*p* = 0.049, $\eta_{p}^{2}$ = 0.808).

### EMG-Torque Relations

**Figure 5:**
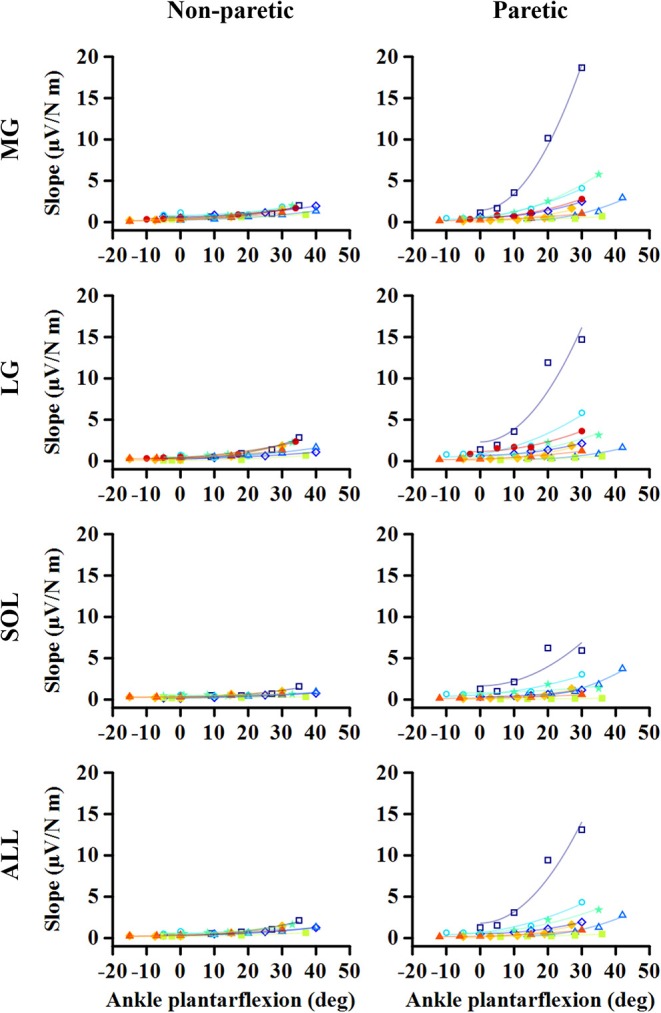
Changes in EMG-torque slope as a function of ankle angle. Note that the effects of changing the ankle angle on the EMG-torque slope are more significant on the paretic side than on the non-paretic side. Markers and lines indicate raw data and fitted results from each individual (in different colors), respectively.

As the ankle angle became more plantarflexed, the slope of the EMG-torque relations increased progressively (Figure 5). Table 1 summarizes the coefficient *a* and *b* for each calf muscle and ALL. For the MG muscle, the coefficient *a* on the paretic side was significantly greater than on the non-paretic side (*p* = 0.008, *d*_*z*_ = 0.549), indicating the greater increase in the slope as the ankle joint becomes plantarflexed. There was no significant difference in the coefficient *b* between sides (*p* = 0.359, *d*_*z*_ = 0.089), indicating that the slope was not significantly different at the maximum DF angles.

**Table 1:** The coefficient *a* and *b* values for the slope of EMG-torque relations (*S*) and ankle plantarflexion angle (*A*). *S* = *aA*^2^ + *b*.

	***a***^#^		***b***	
	**Non-paretic**	**Paretic**	**Non-paretic**	**Paretic**
MG (*n* = 9)	0.655 (0.599–0.790)	2.133^*^ (1.183–3.173)	0.41 (0.28–0.52)	0.35 (0.24–0.49)
LG (*n* = 9)	0.740 (0.465–1.063)	1.657^*^ (1.257–2.505)	0.29 (0.23–0.42)	0.46 (0.19–0.80)
SOL (*n* = 8)	0.258 (0.140–0.376)	0.989^*^ (0.383–2.774)	0.29 (0.22–0.36)	0.36 (0.11–0.61)
ALL (*n* = 8)	0.545 (0.419–0.701)	1.709^*^ (0.886–2.633)	0.33 (0.23–0.42)	0.36 (0.18–0.56)

*MG, medial gastrocnemius; LG, lateral gastrocnemius; SOL, soleus; ALL, average of MG, LG, and SOL*.

# *Actual values are x1,000 smaller than reported*.

* *Significant difference between non-paretic and paretic sides (p < 0.05)*.

Similar results were found in the other muscles. The coefficient *a* on the paretic side was significantly greater than on the non-paretic side (LG: *p* = 0.004, *d*_*z*_ = 0.548; SOL: *p* = 0.008, *d*_*z*_ = 0.802; ALL: *p* = 0.008, *d*_*z*_ = 0.601), whereas there was no significant difference in the coefficient *b* between sides (LG: *p* = 0.250, *d*_*z*_ = 0.501; SOL: *p* = 0.547, *d*_*z*_ = 0.357; ALL: *p* = 0.945, *d*_*z*_ = 0.315).

### Torque-Slope Relations

**Figure 6:**
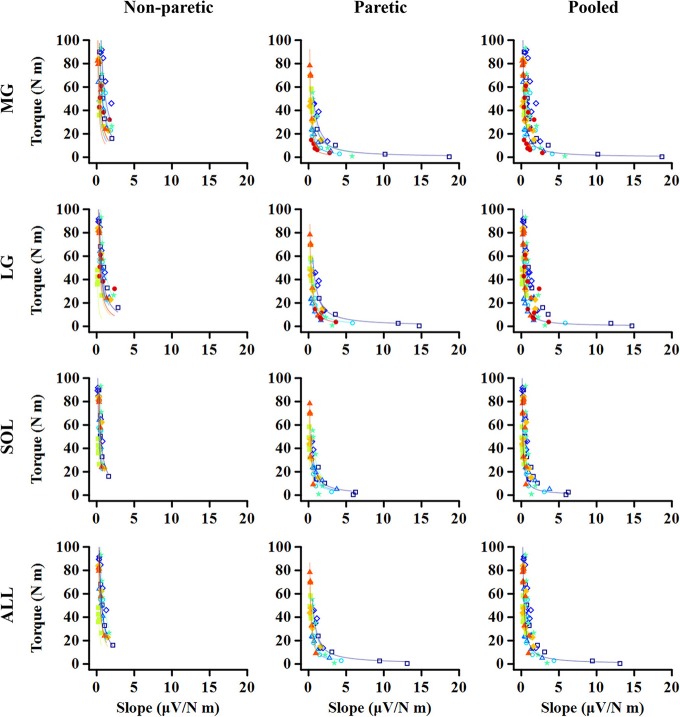
Relationship between maximum torque at each ankle angle and EMG-torque slope at the corresponding angle. Note that the greater slope, the smaller torque. The quicker decay in the torque is shown on the paretic side than on the non-paretic side, whereas there is no significant difference between the paretic and pooled data. Markers and lines indicate raw data and fitted results from each individual (in different colors), respectively.

For all the cases, the maximum voluntary isometric plantarflexion torque was smaller as the slope of the EMG-torque relations was greater (Figure 6). Table 2 summarizes the coefficient *c* for each calf muscle and ALL. The coefficient *c* was significantly smaller on the paretic side than on the non-paretic side (MG: *p* = 0.020, *d*_*z*_ = 1.190; SOL: *p* = 0.016, *d*_*z*_ = 1.326; ALL: *p* = 0.016, *d*_*z*_ = 1.321) except for LG (*p* = 0.098, *d*_*z*_ = 0.627). However, the coefficient *c* for pooled data from both sides was not significantly different with the coefficient *c* on the paretic side for all cases (MG: *p* = 1.000, *d*_*z*_ = 0.053; LG: *p* = 0.074, *d*_*z*_ = 0.622; SOL: *p* = 0.109, *d*_*z*_ = 0.666; ALL: *p* = 0.742, *d*_*z*_ = 0.040).

**Table 2:** The coefficient *c* values for normalized maximum plantarflexion torque at each joint angle (*T*) and slope of EMG-torque relations (*S*). *T* = *cS*^−1^.

	**c**		
	**Non-paretic**	**Paretic**	**Pooled**
MG (*n* = 9)	22.77 (17.51–43.63)	15.10^*^ (11.18–27.00)	17.47
LG (*n* = 9)	26.60 (20.51–30.97)	16.38 (12.53–22.11)	13.33
SOL (*n* = 8)	20.82 (17.38–26.57)	13.99^*^ (10.41–19.67)	10.07
ALL (*n* = 8)	26.99 (22.68–33.70)	14.63^*^ (11.91–26.49)	18.02

*MG, medial gastrocnemius; LG, lateral gastrocnemius; SOL, soleus; ALL, average of MG, LG, and SOL*.

* *Significant difference between non-paretic and paretic sides (p < 0.05)*.

### Correlation Analysis

**Figure 7:**
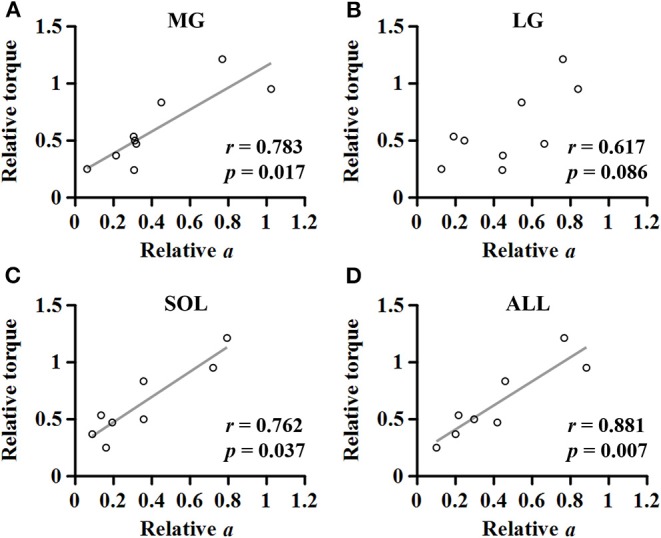
Relationship between the relative maximum torque and the relative coefficient *a* in medial gastrocnemius **(A)**, lateral gastrocnemius **(B)**, soleus **(C)**, and the average of the muscles **(D)**.

A strong linear relationship was observed between the relative coefficient *a* and the relative maximum torque for MG (*r* = 0.783; *p* = 0.017; Figure 7A), SOL (*r* = 0.762; *p* = 0.037; Figure 7C), and ALL (*r* = 0.881; *p* = 0.007; Figure 7D). However, no significant relationship was found for LG (*r* = 0.617; *p* = 0.086; Figure 7B).

**Figure 8:**
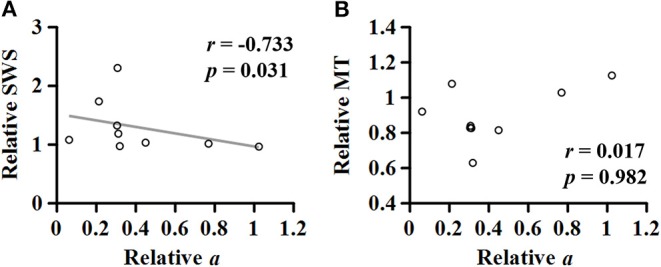
Relationship of the relative (non-paretic/paretic) coefficient *a* with the relative (paretic/non-paretic) shear wave speed (SWS) **(A)** or muscle thickness (MT) **(B)** measured in medial gastrocnemius.

For the MG muscle, the relative coefficient *a* was smaller as the relative SWS measured at the neutral position was greater (*r* = −0.733; *p* = 0.031; Figure 8A). However, the relationship between the relative coefficient *a* and the relative muscle thickness was not significant (*r* = 0.017; *p* = 0.982; Figure 8B).

## Discussion

To summarize, the purpose of this study was to investigate: (1) the effect of changes in ankle joint angles on the muscular contraction efficiency of the calf muscles (i.e., the slope of EMG-torque relations); (2) the relation between the maximum joint torque at each joint angle and the EMG-torque slope at this angle; and (3) the association between the relative coefficient a and the relative muscle thickness or SWS. This is in order to understand the impact of intrinsic muscular changes on muscular contraction efficiency after stroke.

Our results show that the paretic side has a greater slope coefficient *a* (i.e., more increment in the slope as a function of ankle plantarflexion angle) and smaller *c* (i.e., steeper decay in the maximum torque as a function of the slope). There was also a strong linear relationship between the relative joint torque and the relative coefficient a, in the case of MG, SOL, or ALL. For the MG muscles, the relative coefficient *a* (i.e., muscular contraction efficiency) was negatively correlated with the relative SWS (i.e., muscle stiffness).

### The Slope of EMG-Torque Relations Serves as a Measure of Muscular Contraction Efficiency

The greater coefficient *a* on the paretic side indicates that the slope of EMG-torque relations on the paretic side is greater than on the non-paretic side at comparable ankle angles, as also reported earlier (4, 5). Interestingly, the slope of EMG-torque relations has also been used in the field of sports exercise to understand the relative contribution of neural factors and muscle hypertrophy to muscle strength gain in response to muscle strengthening protocol (37). To illustrate, the slope of EMG-torque relations becomes lower as muscle strength increases (37, 38). Although the phenomena induced by training are different (i.e., one is the strength gain due to exercise and the other is strength loss due to injury), the common message may be that the slope of EMG-torque relations is a useful biomarker for muscular contraction efficiency.

Our results showed further that there is a strong relationship between the slope of EMG-torque relations at each joint angle and the normalized maximum joint torque at the corresponding joint angle (Figure 6), suggesting that the absolute slope of EMG-torque relations may be used to estimate the maximum joint torque-capacity. We also found that the relative coefficient *a* showed a strong positive relationship with the relative maximum joint torque (Figure 7), which further suggests that the relative coefficient *a* may be used as a measure of muscular contraction efficiency. This finding is also supported by earlier findings that a greater increase in the cross-sectional area resulted in a greater decrease in the slope of EMG-torque relations (37).

### Effect of Joint Angle Changes on the Slope of EMG-Torque Relations

Based on our finding that the magnitude of RMS EMG at all the measured angles was significantly lower in the MG and LG muscles on the paretic side, the reduction in the magnitude of neural drive seems a primary factor contributing to the reduction in maximum joint torque on the paretic side. In addition, we also found that the effects of changes in joint angle on the slope were more significant on the paretic side than on the non-paretic side (i.e., greater coefficient *a*), although the magnitude of RMS EMG on both sides was not significantly different among the joint angles (i.e., relatively consistent magnitude of neural drive). These findings suggest that muscular changes after stroke, such as muscle atrophy, changes muscle architecture, or changes in material properties, can also contribute to the reduction in muscular contraction efficiency.

The slope increase with increasing ankle plantarflexion (i.e., the coefficient *a*) seems to be associated with active muscle force-length properties. As the practical operating range of calf muscles is usually located in the ascending limb of active force-length curve (27–29), the muscle length becomes shorter as the ankle joint becomes more plantarflexed. Given that the magnitude of force production is smaller at shorter muscle length in the ascending limb, the slope of EMG-torque relations will become greater at a shorter length, characterized here as the coefficient *a* in this study. The greater coefficient *a* on the paretic side can then be explained by an altered active force-length curve after stroke such as narrower width of the curve (19) or by an altered torque-angle curve such as shortened range of the curve (3, 20–22). Since the reduction in the magnitude of peak forces at shorter muscle lengths becomes greater with a narrower curve width, the slope of EMG-torque relations is likely greater on the paretic side than on the non-paretic side.

Our correlation analysis revealed a strong negative relationship between the relative coefficient *a* and the relative SWS measured at the neutral ankle position (Figure 8A), potentially suggesting that the stiffer the muscle, the lower the muscular contraction efficiency. This result can be supported by an earlier simulation study showing that the increased intramuscular fat on the MG muscle resulted in lower force production for a given muscle activation (39). These observations indicate that altered material properties of muscle tissue can also affect muscular contraction efficiency and thus, further investigations are needed to understand how the connective tissues play a role in the voluntary force production.

**Figure 9:**
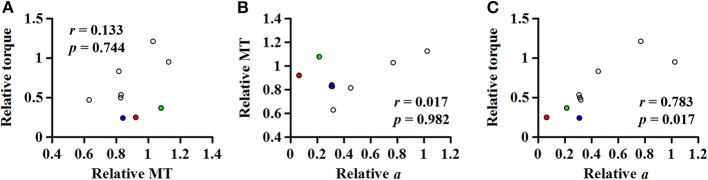
**(A)** Relationship between the relative (paretic/non-paretic) muscle thickness (MT) and the relative maximum torque. Relationship of the relative (non-paretic/paretic) coefficient *a* with the relative MT **(B)** or the relative torque **(C)**. Note that three data points seem as outliers (circles filled with different colors), which had the relatively well-preserved MT but very low relative coefficient *a*, suggesting that chronic stroke survivors may potentially have the different relative contribution of both neural factors and muscular changes to voluntary force generating-capacity after stroke.

We could not establish such a relationship between the relative coefficient *a* and the relative muscle thickness (Figure 8B). To better understand this, given a general agreement that the torque-generating capacity is positively correlated with the muscle thickness or cross-sectional area (40), further correlation analyses were conducted (Figure 9). Although no significant relationship was found between the relative torque and the relative muscle thickness (Figure 9A), there were three data points that seemed as outliers (circles filled with different colors). Interestingly, compared to the non-paretic side, those data points had a relatively well-preserved muscle thickness (Figure 9B) but a considerably affected torque-generating capacity (Figure 9A) as well as a significant effect of ankle joint angle on the slope of EMG-torque relations characterized by the coefficient *a* in this study (Figure 9C). Although it is uncertain, we speculate that those three participants might exhibit a more severe deficit in neural factors rather than in muscular changes. Collectively, it is likely that the relative contributions of neural factors and muscular changes can vary across the clinical spectrum in chronic stroke survivors. Further studies are required to define the relative contribution of both neural factors and muscular changes to voluntary force generating-capacity after stroke.

### Limitations

A significant reduction in the RMS EMGs on the paretic side may sometimes be attributed to non-physiological factors. For example, the relative distance/orientation between the surface electrode location and the innervation zone may be different depending on the ankle angle as well as the contraction intensity (especially in a pennate muscle) (41), ultimately affecting our sEMG signals. This is likely because the pennation angle and fiber length of the MG, LG, and SOL each change with ankle plantarflexion as well as with contraction intensity (42).

Moreover, there have also been reports of altered muscle architecture in the calf muscles after stroke (16, 17, 43, 44), potentially leading to different magnitude of such influences across the participants and across the sides. However, such influences were not distinguishable in our results because the maximum RMS EMG at each ankle angle did not significantly differ on both sides and the magnitude in paretic calf muscles was systematically lower than in contralateral muscles at all measured ankle angle (Figure 3).

Increasing subcutaneous tissue thickness also tends to decrease the sEMG amplitude (45, 46). However, our ultrasound data showed no significant difference in subcutaneous tissue thickness between sides (*p* = 0.055, *d*_*z*_ = 0.517). Moreover, the muscle thickness between sides was not significantly different between sides (*p* = 0.074, *d*_*z*_ = 0.691). Based on these observations, it appears that the decreased RMS EMGs on the paretic side may be likely due to a reduction in neural drive rather than to changes in the subcutaneous tissue/muscle thickness.

It is also important to note that the muscle volume or cross-sectional area may not be a good measure of torque-generating capacity likely due to a loss of active muscle fibers and the substitution of non-contractile tissue (15, 47). Accordingly, more detailed characterization of muscular changes after stroke would help us better understand underlying mechanisms of inefficient muscular voluntary contraction in chronic stroke survivors.

In addition, the magnitude of load-sharing is not readily estimated during voluntary contraction, and thus the average RMS EMG from the calf muscles was used to estimate the overall contribution to the joint torque. Considering that the calf muscle volume does not seem to be affected in a uniform manner after stroke (47), the average RMS EMG may not be accurate enough to represent the overall contribution.

Body mass normalization for torque data is widely accepted, particularly when strength-related outcomes are compared across individuals with varying body masses (48, 49), given a general agreement that muscle strength is positively related with body mass (50). Although we confirmed that our results were not affected by the normalization, there is an issue with such an approach. This is mainly due to non-uniform muscle atrophy across different muscles as well as changes in muscle composition after stroke (47). It is required to establish a reasonable way to compare strength-related outcomes between sides, accounting for muscular changes such as muscle quality (51, 52).

Lastly, our findings are potentially limited by relatively small sample size and by all male participants and thus, special care should be taken to generalize our findings especially due to gender difference in muscle architecture (53).

## Conclusion

In summary, this study examined the EMG-torque relations of calf muscles at different ankle joint angles on both paretic and non-paretic sides in chronic hemispheric stroke survivors. The greater slope of the EMG-torque relations was found on the paretic side compared to the non-paretic side at comparable ankle joint angles (i.e., the greater coefficient *a* on the paretic side), indicating that muscular contraction efficiency may be affected after stroke but in a muscle length-dependent and non-uniform manner. Moreover, the relative coefficient *a* showed a strong relationship with the relative ankle plantarflexion torque or the relative SWS, but was not correlated with the relative muscle thickness. Based on such a discrepancy between the relative reduction in torque-generating capacity and in muscle thickness, our findings suggest that the relative contributions of neural factors and intrinsic muscular changes may vary substantially across the range of chronic stroke survivors. Further studies are needed to explore the relative contribution of both neural factors and muscular changes to voluntary force generating-capacity after stroke.

## Data Availability Statement

The datasets generated for this study are available on request to the corresponding author.

## Ethics Statement

The studies involving human participants were reviewed and approved by Northwestern University's Institutional Review Board. The patients/participants provided their written informed consent for publication in this study.

## Author Contributions

This work was conceived, conducted, analyzed, and authored by JS and WR.

### Conflict of Interest

The authors declare that the research was conducted in the absence of any commercial or financial relationships that could be construed as a potential conflict of interest.
